# RETRACTION: Specific Removal of Nitrite from Lake Urmia Sediments by Biohydrogel Based on Isolated Soy Protein/Tragacanth/Mesoporous Silica Nanoparticles/Lycopene

**DOI:** 10.1002/gch2.70056

**Published:** 2025-10-13

**RETRACTION**: F. Asadzadeh and S. Pirsa, “Specific Removal of Nitrite from Lake Urmia Sediments by Biohydrogel Based on Isolated Soy Protein/Tragacanth/Mesoporous Silica Nanoparticles/Lycopene,” *Global Challenges* 4, no. 12 (2020): 2000061, https://doi.org/10.1002/gch2.202000061.

The above article, published online on 30 September 2020 in Wiley Online Library (wileyonlinelibrary.com), has been retracted by agreement between the journal Editor‐in‐Chief, Mara Staffilani; and Wiley‐VCH GmbH, Weinheim. The retraction has been agreed upon due to inconsistencies observed in the XRD spectra presented in Figure 5. Furthermore, it was identified that an image included in Figure 4 also appears in a previously published article by one of the authors. The authors explained that the XRD spectra in Figure 5 were obtained from an external XRD analysis centre, which was unable to provide the original data. They also explained that the duplication in Figure 4 was the result of an inadvertent copy‐and‐paste error during figure preparation and provided an alternative image. However, due to the unresolved concerns regarding the data, the editors no longer have confidence in the article's results and conclusions. The authors disagree with the retraction.

